# Serum miRNA-21, miRNA-146a and plasma cell free DNA as novel biomarkers for assessing systemic lupus erythematosus activity

**DOI:** 10.1007/s11033-023-08845-z

**Published:** 2023-10-30

**Authors:** Muhammed R.Kh. Ibrahim, Nancy GFM Waly, Hend Moness, Shimaa S. Ahmed, Reham Ibrahem

**Affiliations:** 1 Microbiology and Immunology Department, faculty of pharmacy, Minia University, 61511 Minia, Egypt; 2 Clinical pathology Department, faculty of Medicine, Minia University, Minia, Egypt; 3 Rheumatology, Rehabilitation and physical medicine Department, faculty of Medicine, Minia University, Minia, Egypt

**Keywords:** microRNA, miRNA-21 (MiR-21), miRNA-146a (MiR-146a), Cell-free DNA (cf-DNA), Systemic lupus erythematosus

## Abstract

**Background:**

MicroRNA and cell-free DNA have shown significant correlations with several autoimmune disorders including systemic lupus erythematosus (SLE). SLE has been associated with challenges in determining its activity, so that the need for biomarkers contributing to assessing its activity is emerging. The current study investigated miRNA-21, miRNA-146a and plasma cf-DNA in determination of SLE activity, in addition their association with clinical data including complement factor 3 (C3), complement factor(C4), anti-dsDNA, and other disease activity indices.

**Methods and results:**

Eighty subjects divided into; twenty active patients (with SLE-DAI2K score of 16–18) twenty inactive patients (with SLE-DAI2K score of 1–3), and forty healthy control participants) were included in this study. Serum miR-21, miR-146a, and plasma cf-DNA were quantified by real time PCR and their correlation with clinical data was statistically analyzed. The results demonstrated that active cases have significant upregulation of serum miRNA-21 and plasma cf-DNA. Moreover, miR-21 showed a negative, significant pertaining to C3, C4 and was positively related to Systemic Lupus Erythematosus Disease Activity Index 2 K score (SLE-DAI Index2K score) and Systemic-Lupus-Erythematosus-Disease Activity-Index 2 K activity (SLE-DAI 2 K activity). Also, Active group miRNA-146a was negatively, significantly correlated with C3, as well as a positive significant relationship with SLE-DAI2K score and SLEDAI 2 K activity, in addition to anti DNA Autoantibodies. Furthermore, miR-21 and cf-DNA demonstrated a differential value through Receiver Operating Characteristic (ROC) curve’s study.

**Conclusions:**

the present study illustrated miR-21, miR-146a, and cf-DNA relationship with SLE clinical data. In addition to their potential value in SLE diagnosis, and activity determination.

## Introduction

Systemic lupus erythematosus (SLE) is a prototypic auto-immune condition which is characterized by multi-organ involvement, particularly joints, skin, neurons, and kidneys. SLE has shown to affect women more than men (9:1); furthermore, affecting African women twice more than white women. [1], with worldwide incidence rate of forty SLE patients in each 100 000 [2].

Loss of immune tolerance towards body antigens results in the generation of autoantibodies with high affinities to body nuclear antigens, including histones, ribonucleoproteins, and double stranded DNA (ds-DNA) [3]. Autoantibodies interact with nuclear antigens, forming immune complexes that trigger various immune responses, and contributing to profound inflammation which mediates tissue injury and the presented manifestations [4].

Despite the exact causes of SLE still being elusive, studies showed the contribution of epigenetic factors to disease development including histone modifications, methylation of DNA, modification of histone, and miRNA (non-coding RNA); which their levels of perturbation impact gene expressions without disturbing gene sequences [5]. Micro-RNAs are autogenous non-coding biomolecules with sequences of 18–25 nucleotides. which are either partially or entirely bound to complementary sequences of mRNA and therefore miRNAs are a post-transcriptional regulator of target mRNAs. MiRNAs modulate different biological processes including development, proliferation, and activation of different immune cells including cells, B cells and natural killer cells (NK cells). Thus, disrupted miRNAs are related to the activity of SLE and its progression. Recent studies elucidated the impacts of miRNAs on SLE pathogenesis, through its aberrant profiles contributing to T cells and B cells overresponses and the exaggerated autoantibodies production. through interaction with Lyn tyrosine kinase leading to overactivity of B cells and, consequently, overproducing autoantibodies; the major of SLE pathogenesis [6]. Also, miRNAs affect IFN cascade and dysregulating inflammatory cytokines production via their actions on the transcription factor E2F1. Moreover, dysregulated miRNA levels affect AKT1 in T cells on SLE patients leading to over secretion of inflammatory mediators including IL-4, IL-17, and IFN-γ participating in SLE development [7].

MiRNAs detected Circulating in blood, urine, synovial fluid, and cerebrospinal fluid (CSF) and are associated with disease activity.

MiR-21 regulates gene expression tangled in T cell activation [8]. Hence, miR-21 increased expression in SLE patients renders it to be strongly associated with the disease activity [9]. On the other hand, miR-146a negatively regulates innate signaling cascades, so that decreased expression level of miR-146a is related to SLE exaggerated interferon type I (type I IFN) [10].

Cell-free DNAs (cf-DNAs) are circulating DNA produced through cellular DNA degradation and cell death [11]. Normally cf-DNA circulates in body fluids at a low level, while in SLE, there are excessive secretions of intracellular DNA, either nuclear or mitochondrial DNA as well as hampered clearance as result of deactivated DNases [12]. These free DNA contribute to SLE pathogenesis and are the target for secreted antinuclear autoantibodies forming immune complexes which consequently mediate the profound immune response and inflammation via its interaction with TLR9 which activates antigen-presenting cells with the following T and B cells activation as well as secretion of pro-inflammatory mediators [13].

SLE characterized by rapid progression and development of end organ damage, the features implied the necessity for developing a non-invasive diagnostic tool aiming to early and accurate diagnosis of disease state trying to evade organs involvement, for enhancing patients’ life, overcoming funding issues required in case of disease complications as well as providing therapy monitoring tool and potential management targets.

Recent studies investigated the dysregulated miRNA, cf-DNA levels in SLE patients compared to healthy control. These studies noticed the correlation with routine clinical data, highlighting the potential diagnostic effectiveness of their levels.

Despite routine laboratory data, particularly, anti-ds-DNA (anti-double strand-DNA), anti-nuclear antibodies (ANA), and complement 3 (C3) and 4 (C4), -could help to diagnose and assess disease severity. However, there is an urgent need of an adequate and specific test for confirmation of disease diagnosis, course evaluation, in addition to provide means for SLE prognosis, which opens an eye on the dysregulated level of miRNA and cf-DNA correlating with routine laboratory tests, aiming to standardize a non-invasive diagnostic tool, that helps in early diagnosis to mitigate organs damage, drug monitoring, and dampening SLE mortality rate.

## Materials and methods

### Patients

Before the study, the number of patients required in each group was determined after a power calculation according to data obtained from pilot study. A sample size of 20 patients in each group was determined to provide 80% power for independent samples T-test at the level of 0.05 significance using G Power 3.1 9.2 software. Control group of same number of cases (n = 40) will be included to compare the expression between diseased and health individuals.

The present study conducted during the period from April 2022 to October 2022, in Minia University Hospital. To quantify miRNA and cf-DNA expression and correlate its level to SLE activity we examined eighty subjects precipitated in this study divided into healthy controls and SLE patients all with ages ranging from 20 to 48 years. Furthermore, to compare their dysregulation in both active and inactive SLE patients; the 40 SLE patientswere divided into Group I (active group with SLE-DAI2K score range 8–16) included 20 patients who attended to rheumatology clinic suffering from manifestations suggestive of active SLE stage; Group II (inactive group with SLE-DAI2K score range 1–3) included 20 patients with inactive SLE (patients’ groups were diagnosed applying the Systemic Lupus International Collaborating Clinics’ categorization standards (SLICC 2012) [14]. Control group (III): consisting of 40 age and sex matched individuals. written agreement with full disclosure from all individuals has done, The Research Ethics Committee also authorized this study. (Protocol Approval no. 230,205)

All individuals were subjected to thorough physical examination and extensive medical history. Systemic Lupus Erythematosus Disease Activity Index 2000 (SLE-DAI-2 K) is applied to outline the activity. Our study included patients with SLE with various disease activity states, on the other hand, we precluded patients with other health problems entitled dysregulated miRNA and cf-DNA level involving other autoimmune disorders, acute inflammation, thyroid sickness, diabetes, serious diseases of the liver and kidneys and patients who have experienced a myocardial infarction or strokes. The conditions which might lead to false expression levels, consequently, affect measurements indications and study recommendations and explanations.

### Blood sampling protocol

Aseptically ten millilitres of venous blood were drawn from each person shared in this study, the sample was divided as: (1) two ml placed in ethylene-diamine tetra acetic acid (EDTA)’s vacutainer tube for CBC and for assessment of cf-DNA (2) 800 µ in a tube containing 200 µ of tri-sodium citrate (3.2%) for ESR assay (3) 1.8 ml of blood in vacutainer tube containing 200 µ of tri-sodium citrate (3.2%) for INR measurement (4) The remaining blood were put into an unadorned vacutainer tube. This tube was centrifuged after being allowed to clot at 3000 rpm for 20 min. The expressed serum part of it is used to assay anti-dsDNA, ANA, urea, creatinine, C3, C4 and CRP, While the remaining part is to assay miRNA later.

Blood samples from SLE patients either active group or inactive group as well as control group, drawn during daytime during patients’ hospital visits. Also, patients undergone pre-sample examinations to exclude any interfering conditions such as inflammatory ailments which could affect parameters levels. Collected blood sample from all participants handled, processed in the same manner.

### Routine laboratory investigations

The Sysmex XN-1000™ automated cell counter was used to perform the CBC (Sysmex, Kobe, Japan) [15]. INR was measured by fully automated coagulopathy STA compact, Diagnostic STAGO [16] Urea, creatinine, C3, C4 and CRP anti-dsDNA and ANA by fully automated chemistry analyser, MindrayBS-800, China [17].

### Serum miR-21 and miR-146a quantification

#### microRNA extraction

miRNA extraction was conducted following the protocol manufacturer supplied with miRNeasy extraction kit (Qiagen, Germany) (Cat. No: 217,004). The quality and concentration of extracted miRNA was measured using nanodrop (ND-2000 spectrophotometer, Thermofisher Scientific, USA).

#### Reverse transcription (RT)

RT was conducted utilizing the TaqMan® microRNA RT Kit (Applied Biosystems) (Cat no: 4,366,596), to obtain the cDNA (complementary DNA). reverse transcription process went as follows: half an hour at16 ◦C, then another 30 min at 42 ◦C., followed by 5 min at 85◦C, and holding at 4 ◦C. The produced cDNA was stored at -40 °C until usage.

#### Real-time PCR analysis

TaqMan® MicroRNA Assay kit and TaqMan® Universal Master Mix from Applied Biosystems were utilized to measure the relative expression levels of miR-21 and miR-146a (Cat no: 4,440,043). On a real-time PCR apparatus in step one, the qPCR was conducted (Applied Biosystems) (Cat no: 4,427,975). The following procedures were used to perform qRT-PCR:

- 95 ◦C for 10 min,
- followed by forty series of 15 **s** at 95 ◦C.
- and 1 min at 60 ◦C.

(ΔCT = CT _**miRNA21 or miRNA146−a**_ – CT _**RNU**_), (ΔΔCT = ΔCT_**SLE**_ – ΔCT _**Control**_) and (Folding = 2^− ΔΔCT^) equations were used to compute the expression levels of target miRNA-21 and miRNA146-a. RNU-48 was a reference gene used for normalization. CT gene of interest – CT reference gene) was used to assess the relative expression levels of both miR-21 and miR-146a in serum samples [18].

### Plasma cf-DNA quantification

#### cf-DNA extraction

As instructed by the QIA amp DNA Blood Mini kit’s manufacturer (Qiagen, Germany); cf-DNA was extracted from two hundred µl plasma manually, cf-DNA concentrations were measured by QIAXpert (Qiagen, Germany) after the yield was eluted in 50 µL of elution buffer (Cat. Nos: 51,104).

#### Real-time quantitative PCR

Real-time quantitative PCR applied to measure cf-DNA utilising a Rotor-Gene Q detection equipment and Quanti Tect SYBR Green Master Mix from Qiagen in Germany. (Lot.01065433), (Lot.01018968)

The nucleotides’ sequences of primers were 5′GCGCCGTTCCGAAAGTT3′, for forward primer and 5′CGGCGGATCGGCAAA3′ for reverse primer.

To obtain cf-DNA values, a standard curve was created. The standard curve created using successive dilutions of genomic DNA ranging from 0.00001 to 100 ng/L was used to quantify the absolute DNA concentration [19].

### Statistical analysis

The data were analyzed using the IBM SPSS statistical tool, edition twenty-five. (Armonk, New York, USA: IBM). The data’s normality was evaluated using the Shapiro-Wilk analysis. Parametric quantitative data were indicated as mean ± Standard Deviation and range. On contrary, the quantitative non-parametric data were expressed by median (IQR), moreover, the qualitative information indicated by both number and percentage.

The three groups’ quantitative parametric data were assessed employing a One-Way ANOVA test and a post hoc Tukey’s analysis between each two groups, while the quantitative non-parametric data were tested using Kruskal Wallis test, Mann Whitney test is then conducted between each two groups. Moreover, Using the Chi-square test or Fisher’s exact test, categorical variables were compared.

Using Pearson’s correlation, the relationship between the variables was found. To determine the area under the curve (AUC), ideal cutoff point, sensitivity, specificity, positive and negative predictive values, and accuracy of variables predicting cases, ROC curve analysis was conducted. Statistical significance was defined as a p-value < 0.05.

## Results

### Participant’ data

Our findings showed insignificant age and sex difference for all groups. Concerning SLEDAI 2 K activity, in group (I) there was 45% moderate, 55% severe cases compared to group (II) where 95% mild and 5% moderate. SLE-DAI2K score range 8–16 in Group I while 1–3 in Group II. (Table 1)

**Table 1:** Demographic data for Group I, Group II and Group III as well as disease activity parameters including SLE-DAI2K score and SLEDAI 2Kactivity

Demographic data	Parameters	active (Group I)	inactive (group II)	Control (group III)	P value			
					Among 3 groups	Active vs. Inactive	Active vs. Control	Inactive vs. Control
		N = 20	N = 20	N = 40				
Subjects’ Age	*Range* *Mean ± SD*	(23–47) 35.5 ± 8.6	(20–48) 30.9 ± 8.3	(22–46) 33.1 ± 8.1	0.225	0.215	0.567	0.596
SLE-DAI2K score	*Median* *IQR*	12 (8–16)	2 (1–3)		< 0.0001*			
SLEDAI 2Kactivity	Mild Moderate Severe	0(0%) 9(45%) 11(55%)	19(95%) 1(5%) 0(0%)		< 0.0001*			
Sex	Female Male	19 (95%) 1 (5%)	18(90%) 2(10%)	36(90%) 4 (10%)	*0.26*			

Data are indicated as number (%) or mean ± SD or median (25th–75th)

### Participants’ routine lab investigations

There was a significant difference in Hb concentration when comparing Group I and Group II versus Group III (p ≤ 0.001), Moreover, Hb concentration did not significantly differ between group I and group II. Total leukocytic count (TLC) showed only significance between group I and III. Moreover, there were insignificant differences between all subjects in blood urea, serum creatinine, INR, lymphocytic count, and platelets count. Finally, ESR level showed a significant difference between all groups and between Group I versus Group III and Group II versus Group III (Table 2).

**Table 2:** Routine laboratory data measured for Group I and Group II compared to Group III.

Laboratory data	Parameters	Active patients (Group I)	Inactive patients (group II)	Control (group III)	The p value			
					Among 3 groups	Active vs. Inactive	Active vs. Control	Inactive vs. Control
		N = 20	N = 20	N = 40				
Hb	*Range* *Mean ± SD*	(8.7–13.5) 10.8 ± 1.5	(7.6–13.0) 10.6 ± 1.7	(12.1–16) 14.2 ± 1.1	< 0.001	0.925	< 0.001	< 0.001
TLC	*Median* *IQR*	5.1 (4-7.1)	6 (3.7–10)	7 (6–9)	< 0.001	0.264	< 0.001	0.238
Lymphocytic count	*Median* *IQR*	26.3 (17.5–34.2)	34 (14-36.1)	30 (25–37)	0.655	0.998	0.806	0.718
Platelet count	*Range* *Mean ± SD*	(118–426) 282.2 ± 116	(102–381) 246 ± 87	(151–446) 280 ± 97	0.171	0.509	0.889	0.109
ESR	*Median* *IQR*	45 (30–70)	60 (55–70)	5 (3–7)	< 0.001	0.955	< 0.001	< 0.001
INR	*Range* *Mean ± SD*	(1–2) 1.1 ± 0.2	(1–1) 1 ± 0.06	(0.9-1) 1 ± 0.07	0.166	0.590	0.442	0.691
Urea	*Range* *Mean ± SD*	(16–36) 27 ± 5.5	(16–44) 25.7 ± 9	(20–45) 28.7 ± 6.3	0.413	0.793	0.814	0.509
Creatinine	*Range* *Mean ± SD*	(0.5–1.2) 0.9 ± 0.12	(0.5-1.6) 0.87 ± 0.3	(0.6-1.5) 1 ± 0.2	0.132	0.939	0.206	0.275

Data is presented as median or the mean ± SD (25th–75th)

### Inflammatory and immunological indicators

Moreover, inflammatory, and immunological markers (Table 3) same as CRP was positive in 35% of Group I compared to 30% of Group II. Serum level of both C3 and C4 revealed statistical difference when comparing all groups with each other (p = < 0.001). Anti-ds-DNA was positive in 80% of Group I while only 25% of Group II were positive. 85% of patients had positive ANA in Group I in contrast to 80% of Group II.

**Table 3:** Measured Inflammatory markers in Group I and Group II compared to Group III.

Inflammatory markers	Parameters	Active (I)	inactive (II)	Control (III)	P value			
					Between all groups	Active vs. Inactive	Active vs. Control	Inactive vs. Control
		N = 21	N = 14	N = 35				
CRP	Positive Negative	7(35%) 13(65%)	6(30%) 14(70%)	0(0%) 40(100%)	< 0.001	*0.736*	< 0.0001*	< 0.0001*
ANA	Positive Negative	17(85%) 3(15%)	16(80%) 4(20%)	0(0%) 40(100%)	< 0.001	*0.681*	< 0.0001*	< 0.0001*
Anti-dsDNA	Positive Negative	16(80%) 4(20%)	5(25%) 15(75%)	0(0%) 40(100%)	< 0.001	< 0.0001*	< 0.0001*	< 0.001*
C3	*Range* *Mean ± SD*	(28–44) 33.35 ± 4.9	(60–112) 90.05 ± 15.2	(102–178) 142.4 ± 23.9	< 0.001	< 0.001	< 0.001	< 0.001*
C4	*Range* *Mean ± SD*	(5.1–15) 12.15 ± 2.1	(12.3–35) 24.9 ± 6.3	(13–40) 32 ± 7.6	< 0.001	< 0.001	< 0.001	< 0.001

Data are expressed as number (%) or mean ± SD

### MiRNA-21 relative expression and its relationship with clinical data

The levels of miRNA-21 were (9.67 ± 1.2-fold change) in Group I, (3.4 ± 0.76-fold change) in Group II and (2.2 ± 0.2-fold change) in Group III. Moreover, findings showed a significant variation among studied subjects (**p** less than 0.001).

miRNA-21 relative expression in Group I shown to be positively correlated with ESR level (r = 0.337), Anti-dsDNA (r = 0.304), in addition to significantly correlated with SLE-DAI2K score and SLE-DAI2K activity (r = 0.447). while negatively correlated to serum C4 (r = -0.425), and negatively significant correlated to serum C3 (r = -0.587).

Group II there were only fair negative correlation of miRNA 21 relative expression with ESR (r= -0.395*) and positive correlation with serum C3, C4 (r = 0.422*, 0. 344* respectively) (Table 4).

**Table 4:** MiRNA-21 Relative expression correlations with clinical Data

miRNA21					
Clinical data	Active subjects (n = 20)			Inactive subjects (n = 20)	
	r	p		R	p
ESR	0.337**	0.147		− 0.395**	. 085
C3	− 0.587**	0.006*		0.422**	. 064
C4	− 0.425**	0.062		. 344**	. 138
SLE-DAI2K score	0.447**	0.048*		0.073	0.760
Anti-ds-DNA	0.304**		0.193	-0.190	0.422
SLE-DAI 2 K activity	0.447**		0.048*	0.073	0.760

^*^P value < 0.05, **r > 0.3

### MiRNA-146a relative expression and relationship with clinical data

Group I miR-146a serum levels were (5.79 ± 0. 66)-fold increase. Additionally, the serum miR-146a level in Group II possessed a (7.43 ± 2.12-fold increase). Both patients’ groups showed significant higher levels than Group III (p < 0.001).

MiRNA-146a in group I showed to be negatively significantly pertained to C3 level (r of − 0.432). While miRNA-146a was positively correlated with ESR level (r = 0.337*) and significantly positively pertaining to Anti-dsDNA (r = 0. 441). In group II miRNA 146a showed negative fair correlation with ESR (r = -0.312). Serum C3 level showed to be of positive correlation (r = 0. 348) with miRNA-146a (Table 5).

**Table 5:** MiRNA-146a Relative expression relationship with clinical Data

miRNA 146a					
Clinical data	Active subjects (n = 20)			Inactive subjects (n = 20)	
	r	p		r	p
ESR	0.337**	0.147		− 0.312**	. 180
C3	− 0.432**	. 057*		. 348**	. 132
C4	0.176	. 459		. 137	. 565
SLE-DAI2K score	0.024	0.024*		− 0.252	. 284
Anti-ds-DNA	0.441**		. 052*	− 0.238	. 311
SLE-DAI 2 K activity	0.024		0.024*	− 0.252	. 284

^*^P value < 0.05, **r > 0.3

### Expression of cf-DNA and its correlation with clinical data

Group I cf-DNA plasma levels were (16.94 ± 1.85)-fold increase. Additionally, the plasma cf-DNA level in Group II possessed a (11.38 ± 2.05-fold increase), compared to (9.73 ± 1.1- fold increase) in Group III. Plasma level of cf-DNA expressed significant difference when comparing all groups to each other (p = < 0.001).

Cf-DNA level in Group I shown to be positively correlated with ESR (r = 0.412), SLE-DAI2K score and SLE-DAI2K activity (r = 0.326). C3 (r= -0.305) and C4(r= -0.395) were negatively correlated. While in Group II, cf-DNA showed a positive correlation with SLE-DAI2K score and SLE-DAI2K activity (r = 0.326). in contrast to a negative correlation with C3 (r= -0.316) (Table 6).

**Table 6:** Cf-DNA expression correlations with clinical laboratory data

cf-DNA				
Clinical data	Active subjects (n = 20)		Inactive subjects (n = 20)	
	r	p	R	p
ESR	0.412**	. 071	− 0.003	0.989
C3	− 0.305**	0.191	− 0.316**	0.175
C4	− 0.395**	0.085	0.111	0.640
SLE-DAI2K score	0.326**	0.161	0.326**	0.160
Anti-ds-DNA	− 0.079	0.741	0.052	0.828
SLE-DAI 2 K activity	0.326**	0.161	0.326**	0.160

**r > 0.3

### Plasma cfDNA and serum miR-21 showing diagnostic significance in SLE patients

The ROC (Receiver Operating Characteristic) curve was made using data of serum level of miRNA21 and plasma level of cf-DNA from Group I and Group II to estimate its the adequacy in SLE activity assessment.

**Fig. 1:**
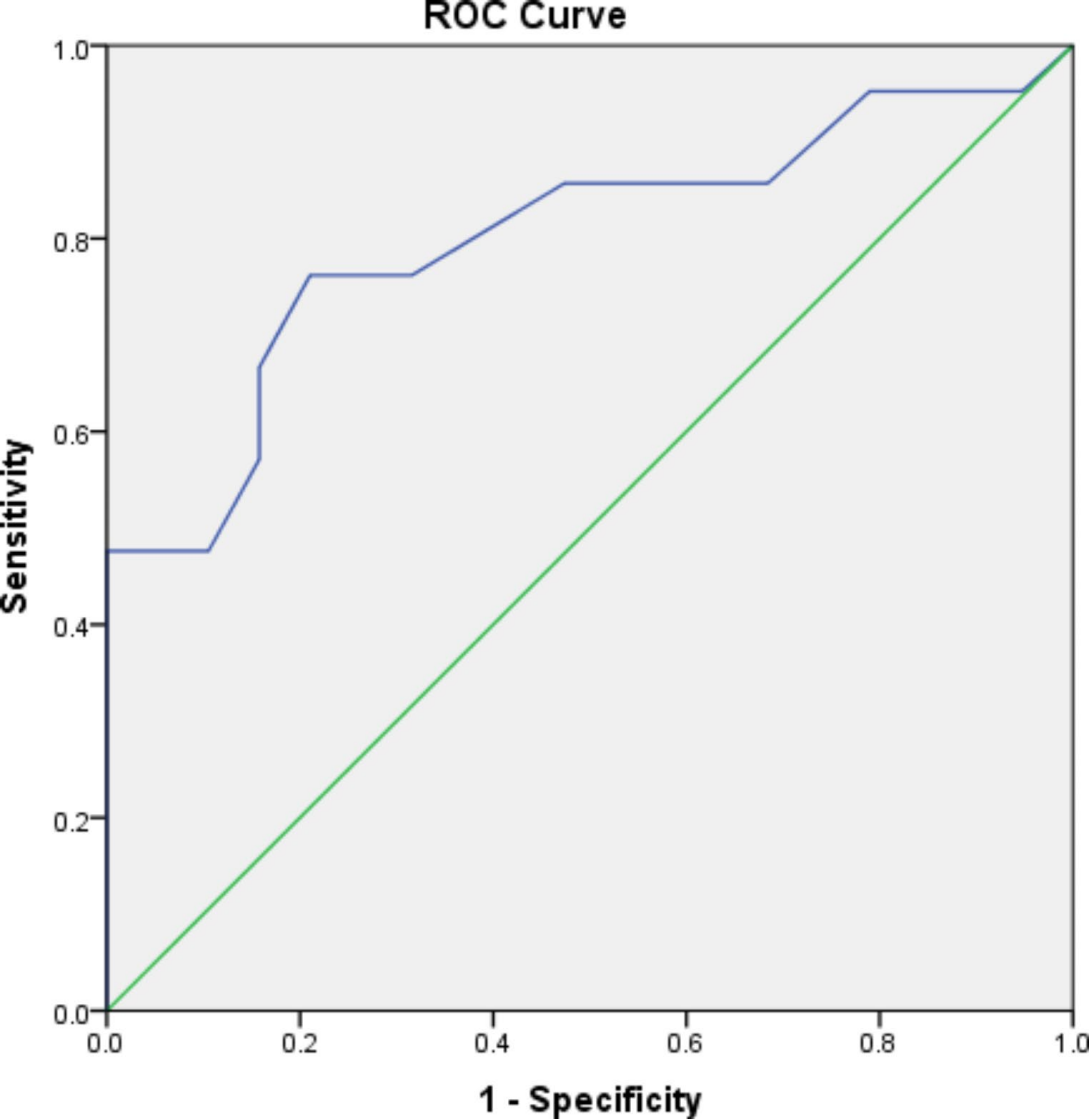
Receiver Operating Characteristic (ROC) curve for miRNA 21

ROC curve presented miRNA-21 with a cut off value of **≥** 3.95 with AUC score of 0.801 (**p** score less than 0.001) compared to Group III. analysis showed a detection sensitivity percentage with 85.71% as well as 52.63% as specificity percentage, PPV with 66.67%, a NPV of 76.92% and a differential accuracy of 70.0% (Fig. 1).

**Fig. 2:**
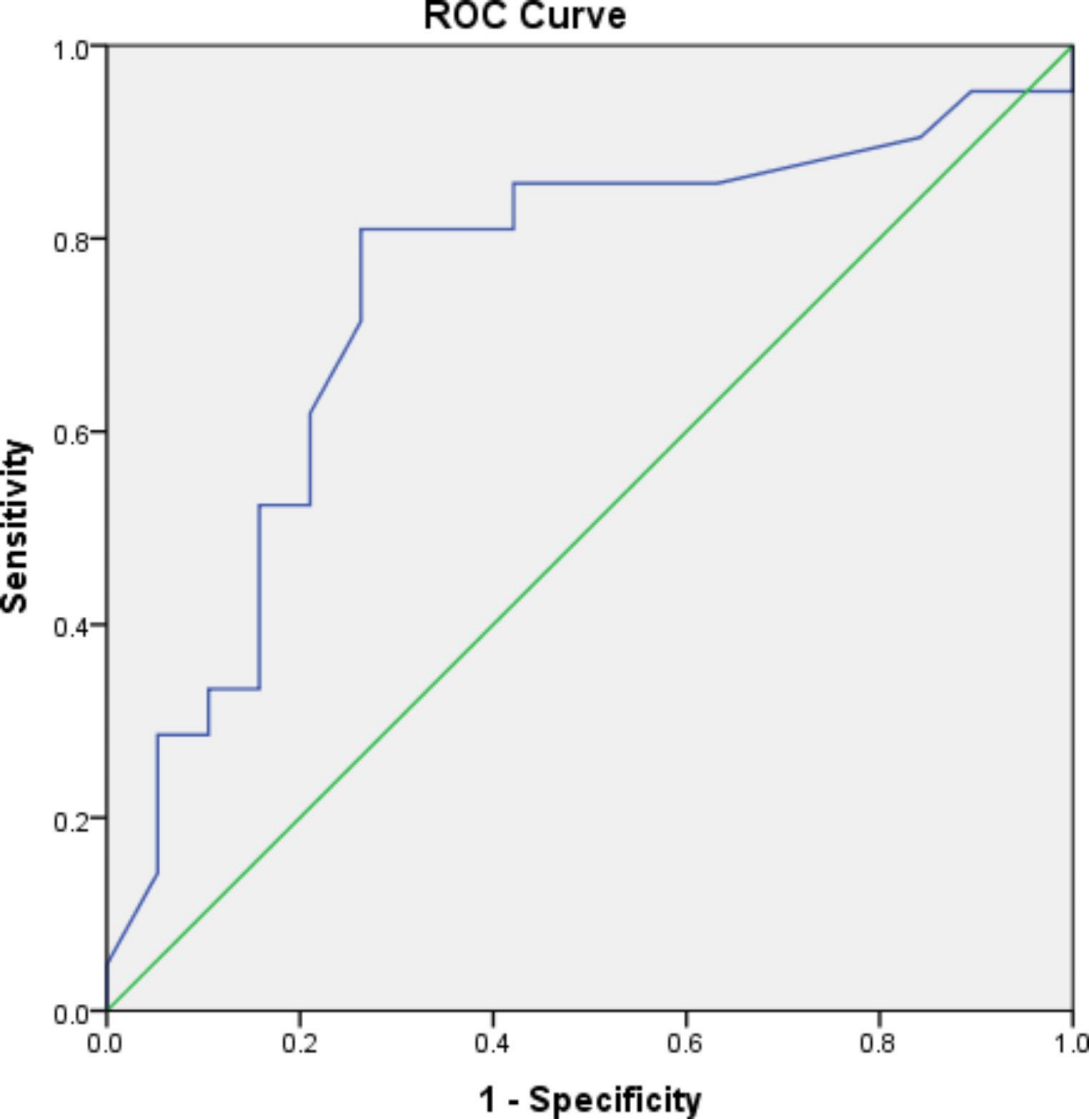
Receiver Operating Characteristic(ROC) curve for cf-DNA.

Furthermore, cf-DNA showed a cut off value of **≥** 14.35 with score of AUC of 0.743 (p score equals 0.009) comparing to Group III. analysis showed a sensitivity percentage of 80.95%, as well as 73.68% as specificity percentage, 77.27% as PPV proportion, a NPV score with 77.78% in addition to differential accuracy score with 77.50% (Fig. 2).

### The intercorrelation of Cf-DNA, miRNA 21, and miRNA146a

**Fig. 3 In the PDF, please increase the size of fig. 3 as it is not readable:**
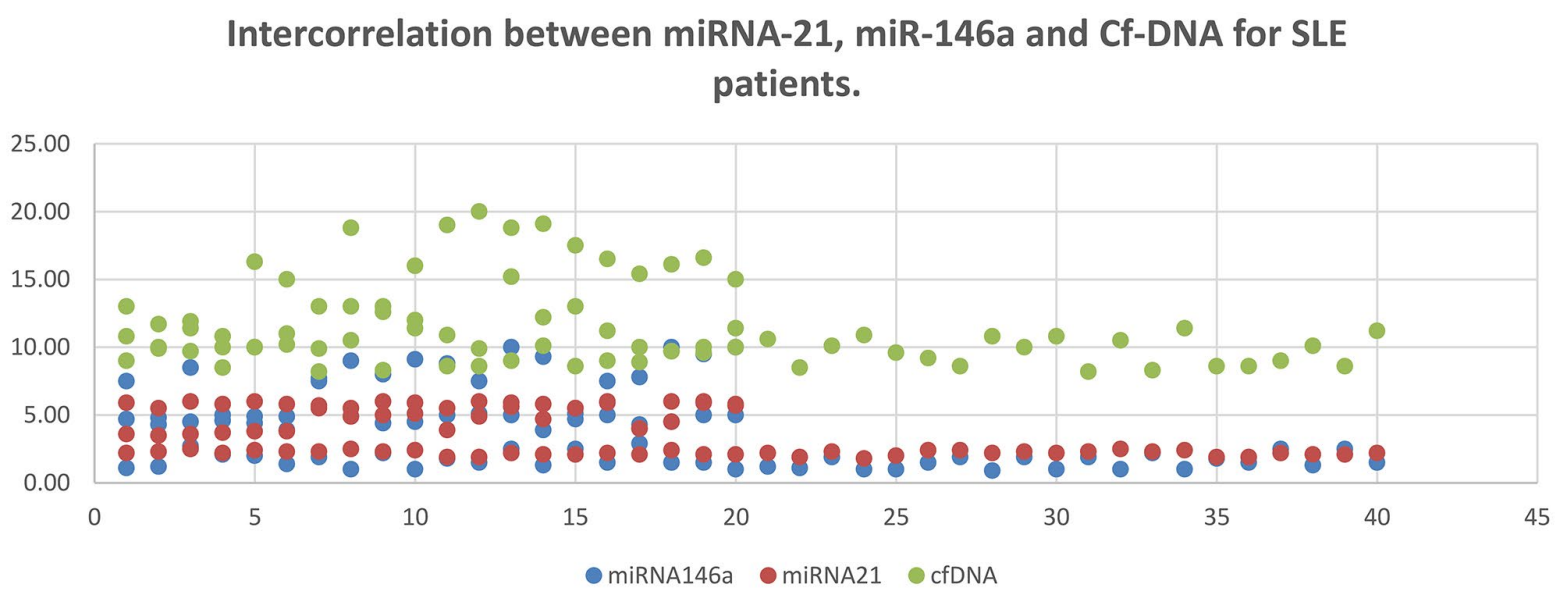
the intercorrelation between miRNA-21, miR-146a and Cf-DNA for SLE patients

all disease subjects’ results showed that miRNA-21 possess significant negative correlation with miRNA-146a (p = 0.01, r = **-**0.404) and of positive considerable correlation with cf-DNA (p **<** 0.001, r = 0.786). Moreover, miRNA146a showed significant negative correlation with both miRNA21 and cf-DNA (p score equals to 0.01, r = − 0.404) and (p score of 0.007, r = − 0.422) respectively (Fig. 3).

## Discussion

SLE is a long-term autoimmune condition hinging on disabled immune tolerance, the overproduction of sustained antinuclear antibodies, and excessive inflammatory mediators. Leading to clinical symptoms varying from minor symptoms to serious illnesses [4]. With the concomitant lack of an accurate tool for identifying disease activity and the difficulty to evade disease flares. Furthermore, the overlapping of the disease with other autoimmune diseases renders it difficult to disease diagnosis [20].

Since the patients diversly manifest constitutional alerts, hematological manifestations, arthritis, skin involvement, lupus nephritis and central nervous system involvement [2]. either the early detection of the disease or monitoring disease activity help to evade such complications with subsequent patients’ health improvement.

Our study involved 40 SLE patients with age ranges from 20 to 48 with 92% of patients being women which are backed to sex hormones as well as the effects of X-chromosome [21]. Involved patients were grouped -based on disease activity classification using SLEDAI [21]- into Group I with 55% with severe disease and 45% of subjects with moderate severity. And group II with 95% of subjects with mild activity and 5% were moderately severe.

Shamim et al. reported a substantial disease activity correlation with hematological manifestations including Hb, ESR, and TLC, which agrees with our findings for the Group I [22]. While Group II encountered a significant decrease in Hb concentration, a main effect in SLE [23]. Moreover, Group II showed a considerable increase in ESR, which could be interpreted through the encountered inflammation for all SLE cases [24].

SLE pathogenesis involves the consumption of complement proteins leading to significant downregulation, in accordance with our observations for SLE groups, albeit there was an excessive decrease for Group I which could be interpreted via the profound inflammation presented in Group I [21] [24]. Since SLE experiences upregulated anti-ds-DNA levels, our results presented upregulation of Anti-ds-DNA for most Group I (80%), besides moderate subjects of the Group II (25%), supporting studies that reported its association with disease activity [25]. The results also showed a rise in ANA for both Group I and II. In accordance with observations made by H. Li et al. [26]. CRP is a sensitive tool for detecting ongoing inflammation and consequently, disease activity, which agrees with the findings that showed a positive significant detection of CRP in active cases. Nevertheless, CRP is a nonspecific biomarker as it is implicated in various diseases, not only SLE [24]. Overall, inflammatory biomarkers are significantly correlated with experienced inflammation in SLE, which explains the significant increase in active patients. However, it is also elevated in other autoimmune diseases, infections, and other inflammatory conditions. Therefore, it could not be depicted as specific SLE diagnostics or sole disease activity markers. Consequently, the correlation of miRNA and cf-DNA with clinical data might help to more sensitive and specific diagnosis as well as prediction of disease course.

Several studies reported contributions of miR-21 to T cell activation [6], Dendritic cells (DCs) differentiation through regulation of the Protease for programmed cell death4 (PDCD4) and the stromal antigen 2 (STAG2), and granulocyte activation [27] resulting in excessive inflammatory mediators and enormous cell death which augment the association of miR-21 to SLE activity [28]. Elucidating findings showed significant upregulation of miRNA-21 in both patients. Furthermore, there was miR-21 overexpression in active cases more than inactive cases (9.67 times in active cases/3.4 times in inactive cases); which agrees with findings reported by Allawe, Abed, Abdullah [29].

Group I miR-21 showed a fair positive correlation with SLE-DAI scores and SLE-DAI 2 K activity (r = 0.447, p = 0.04), while the relationship was poor in Group II(r = 0.07). Furthermore, there was a fair association of miR-21 with Anti-dsDNA and ESR levels for Group I while weak for Group II consistent with Guo et al. [30]. Moreover, Group I miR-21 showed a negative, significant relationship to C3 (r = − 0.587, p = 0.006) as well as C4 (r = − 0.425, p = 0.062), which is elucidated since the overproduced autoantibodies consume the complements via stimulation of complement activation classical pathway. These autoantibodies are produced through downregulating PDCD4 by miR-21 and augmenting the expression of interleukin 10(IL-10), which in turn stimulates B cell differentiation [31].

miR-146a contributes to the immunological responses in SLE; via upregulating IFN type I, TLRs, and the gene-I-like receptors induced by retinoic acid (RLRs) by enhancing the innate immune cells [32].

Our findings showed significantly upregulated miR-146a for all patients which agrees with findings observed by Labib et al., [33]. while contradicting findings presented by Nagy et al., who noticed the downregulation of serum miR-146a [34]. This is interpreted via Th1’s miR-146a overexpression contradicting Th2 downregulation with correspondence to its level in undifferentiated cells [35].

The findings showed the upregulation of miR-146a in Group II (7.43 ± 2.12times) was more than in Group I (5.79 ± 0.66times). Augmenting studies reported miR-146a’s negative association with SLE activity [36], which could be understood by IFN type I stimulation of monocyte chemotactic protein-induced protein1 hindering miR-146a maturation with subsequent overexpression of genes responsible for inflammatory actions in SLE [37], so Group I, who experiences a profound inflammation possess lower miR-146a than Group II.

Also, miR-146a in Group I was significantly and negatively related to C3(r = − **0.4**32, p = 0.057), which explained as the more inflammation and disease activity, the lower miR-146a. while miR-146a possessed a significant, and positive relationship with Anti-ds-DNA (r =. 441, p =. 052) in accordance with Li et al. [38] which is elucidated based on increased activity accompanied by low miR-146a as well as excessive autoantibodies including Anti-dsDNA. While, the Group II, miR-146a is positively linked to C3 (r = 0. 348) and negatively with ESR (r=-0.312) which agrees with Tawfik et al. 2019 [39].

Moreover, our findings revealed a significant upregulation of cfDNA for all patients, which agrees with Hendy et al.,[413]. The upregulation could be explained by ineffective elimination of the profound necrosis and apoptosis presented in SLE [40], the excretion of DNA from neutrophils extracellular traps as well as disabled degradation presented in SLE [41]. Furthermore, the neutrophil extracellular traps also incriminated in inflammatory responses and contribute to vascular inflammation, atherogenesis as well as autoinflammatory cases [42]. Furthermore, findings showed noticed cf-DNA upregulation in Group I (16.94 folds) more than Group II (11.38 folds) which augments the assumption of its correlation with SLE activity.

Additionally, Group I showed cf-DNA with a fair negative correlation with C4 while both groups are negatively correlated with C3. This supports the assumption of cf-DNA association with SLE activity and the encountered excessive autoantibodies that enhance complement consumption. In accordance with observations made by Tug et al. [43]. while contradicting results made by Gerli R. which identified no relation of cf-DNA with either C3 or C4 [44]. Furthermore, cf-DNA levels show a fair positive relationship with ESR, SLE-DAI2K score, and SLEDAI 2 K activity which augment the possible roles of cf-DNA in SLE development. This finding agrees with the results presented by Xu. et al. [45]. Albeit contradicting Tug et al. findings which state no correlation between cf-DNA and activity parameters [43]. Furthermore, cf-DNA was correlated with Anti-dsDNA which agrees with Hendy et al. [46] which augments the possible vital role of anti-dsDNA in SLE pathogenesis for its predisposing inflammatory responses [47].

Moreover, our study revealed that miR-21 possesses a good diagnostic value (AUC = 0.801). with 85.71% sensitivity and 52.63% specificity. In agreement with Zheng et al. [48] who revealed that miRNA-21 of AUC value was 0.8281. Furthermore, cfDNA shows a fair diagnostic value (AUC = 0.743) with 80.95% sensitivity and 73.68%. specificity. in agreement with Giaglis et al. [49] who showed cfDNA with an AUC value equal to 0.7. Conversely, miR-146a showed insignificant diagnostic value with recommendations for further investigations using more samples.

For our knowledge, previous studies individually investigated the expression of various miRNAs and cf-DNA levels. But neither correlating miRNAs with cf-DNA levels nor with clinical data; the notification derived our study to investigate.

The implementation of miRNAs and cf-DNA in SLE; is likely to provide efficient reliable auxiliary diagnosis biomarkers as well as outline clear patterns for treatment decisions. Our study revealed a moderately significant relationship of miR-21 with cf-DNA (r = 0.786, **p** < 0.001); indicating their positive roles in SLE activity, while a considerable negative association of miR-21 with miR-146a and (r = − 0.404, p = 0.01); indicating the miR-21 clear correlation with the activity of SLE. While cf-DNA had a negative fair significant relationship with miR-146a (r = -0.422, p = 0.007) augmenting the assumption of inversed correlation between miR-146a with SLE activity. Moreover cf-DNA via its significant correlation with both miR-21 and miR-146a; provides a direct correlation with SLE activity.

The variability between miRNA and cfDNA levels among SLE patients in current study and other studies may be due to the patient’s ethnicity variations and different environmental factors exposure [50]. as well as sample sources, sample size, lifestyle, and dietary habits which might elucidate the probable causes of variable results [51]. Our findings strongly suggest miR-21 and cfDNA could be of potential diagnostic value for SLE disease through their involvement in SLE pathogenesis and thus expecting the disease activity and severity. The impacts that virtually could contribute to hamper disease complications, monitoring disease activity status with subsequent therapeutic interventions, and giving insights into potential therapeutic targets.

## Conclusion

SLE is an autoimmune disorder marked by poor prognosis and patients might experience several life-threatening complications particularly cardiovascular, renal, and neural involvement. Therefore, there is a crucial demand for specific and accurate diagnostic tools and treatment targets to improve patients’ lives.

The current study quantified serum miRNA-21, miRNA-146a, and plasma cfDNA SLE expression. The findings showed significant correlations between miR-21, miRNA-146a and cfDNA and clinical biochemical markers particularly those reflecting SLE activity status. As well as miR-21 and cf-DNA level revealed a good diagnostic value particularly when coupled to other clinical data. Consequently, it could be invested as efficient prognostic tools for SLE disease. Furthermore, the correlation of three parameters could provide a great aptitude biomarker for SLE diagnosis and prognosis.

## Data Availability

This article’s published data set contains all information created or analyzed during this investigation.

## Abbreviations

miR-21: MicroRNA-21
miR-146a: MicroRNA-146a
Cf-DNA: Cell free DNA
SLE: Systemic-Lupus-Erythematosus
C3: Complement 3
C4: Complement 4
Anti-dsDNA: Anti-double strand DNA
SLE-DAI: 2 K score Systemic-Lupus-Erythematosus -Disease Activity- Index 2 K score
SLEDAI: 2 K activity Systemic-Lupus-Erythematosus-Disease Activity-Index 2 K activity
ROC: Curve Receiver Operating Characteristic (ROC) curve
Type I IFN: Type I Interferon
TLR: Toll Like Receptor
ANA: Anti-Nuclear-Antibody
SLICC: Systemic-Lupus-International-Collaborating Clinics
CBC: Complete-Blood-Count
CRP: C-Reactive-Protein
ESR: Erythrocyte-Sedimentation-Rate
INR: International Normalized Ratio
EDTA: Ethylene-Diamine Tetra Acetic acid
cDNA: Complementary DNA
RT-PCR: Real-Time PCR
AUC: Area Under the Curve
Hb: Hemoglobin
TLC: Total Leukocyte Count
PPV: Positive Predictive Value
NPV: Negative Predictive Value
DCs: Dendritic cells
PDCD4: Programmed-Cell-Death-Protein 4
STAG2: Stromal-Antigen-2
IL-10: Interleukin 10
RLRs: Retinoic-acid-inducible gene-I-Like-Receptors
